# Multi-dose Oral Ondansetron for Pediatric Gastroenteritis: study Protocol for the multi-DOSE oral ondansetron for pediatric Acute GastroEnteritis (DOSE-AGE) pragmatic randomized controlled trial

**DOI:** 10.1186/s13063-020-04347-6

**Published:** 2020-05-27

**Authors:** Stephen B. Freedman, Sarah Williamson-Urquhart, Anna Heath, Petros Pechlivanoglou, Gareth Hopkin, Serge Gouin, Amy C. Plint, Andrew Dixon, Darcy Beer, Gary Joubert, Christopher McCabe, Yaron Finkelstein, Terry P. Klassen, on behalf of the KidsCAN-Pediatric Emergency Research Canada (PERC) Innovative Pediatric Clinical Trials DOSE-AGE Study Group

**Affiliations:** 1 Sections of Pediatric Emergency Medicine and Gastroenterology, Departments of Pediatrics and Emergency Medicine, Alberta Children’s Hospital, Alberta Children’s Hospital Research Institute, Cumming School of Medicine, University of Calgary, Calgary, AB Canada; 2 Alberta Children’s Hospital, Cumming School of Medicine, University of Calgary, Calgary, AB Canada; 3 The Hospital for Sick Children, Division of Biostatistics, Dalla Lana School of Public Health, University of Toronto, Toronto, ON Canada; 4 Department of Statistical Science, University College London, London, UK; 5 The Hospital for Sick Children, Institute for Health Policy Management and Evaluation, University of Toronto, Toronto, ON Canada; 6 Institute of Health Economics, Edmonton, AB Canada; 7 Departments of Pediatric Emergency Medicine and Pediatrics, CHU Sainte-Justine, Université de Montréal, Montreal, QC Canada; 8 Children’s Hospital of Eastern Ontario, Departments of Pediatric and Emergency Medicine, University of Ottawa, Ottawa, ON Canada; 9 Stollery Children’s Hospital, Department of Pediatrics, University of Alberta, Women and Children’s Health Research Institute, Edmonton, AB Canada; 10 Max Rady College of Medicine, Pediatrics and Child Health, Rady Faculty of Health Sciences, University of Manitoba, and the Children’s Hospital Research Institute of Manitoba, Winnipeg, MB Canada; 11 Division of Paediatric Emergency Medicine, Department of Paediatrics, Children’s Hospital LHSC, Western University, London, ON Canada; 12 Institute of Health Economics and the Department of Emergency Medicine, University of Alberta, Edmonton, AB Canada; 13 Divisions of Emergency Medicine and Clinical Pharmacology and Toxicology, Department of Paediatrics, Hospital for Sick Children, University of Toronto, Toronto, ON Canada

**Keywords:** Ondansetron, Child, Vomiting, Gastroenteritis, Dehydration, Emergency service, hospital, Randomized controlled trial

## Abstract

**Background:**

There are limited treatment options that clinicians can provide to children presenting to emergency departments with vomiting secondary to acute gastroenteritis. Based on evidence of effectiveness and safety, clinicians now routinely administer ondansetron in the emergency department to promote oral rehydration therapy success. However, clinicians are also increasingly providing multiple doses of ondansetron for home use, creating unquantified cost and health system resource use implications without any evidence to support this expanding practice.

**Methods/design:**

DOSE-AGE is a randomized, placebo-controlled, double-blinded, six-center, pragmatic clinical trial being conducted in six Canadian pediatric emergency departments (EDs). In September 2019 the study began recruiting children aged 6 months to 18 years with a minimum of three episodes of vomiting in the 24 h preceding enrollment, <72 h of gastroenteritis symptoms and who were administered a dose of ondansetron during their ED visit. We are recruiting 1030 children (1:1 allocation via an internet-based, third-party, randomization service) to receive a 48-h supply (i.e., six doses) of ondansetron oral solution or placebo, administered on an as-needed basis. All participants, caregivers and outcome assessors will be blinded to group assignment. Outcome data will be collected by surveys administered to caregivers 24, 48 and 168 h following enrollment. The primary outcome is the development of moderate-to-severe gastroenteritis in the 7 days following the ED visit as measured by a validated clinical score (the Modified Vesikari Scale). Secondary outcomes include duration and frequency of vomiting and diarrhea, proportions of children experiencing unscheduled health care visits and intravenous rehydration, caregiver satisfaction with treatment and safety. A preplanned economic evaluation will be conducted alongside the trial.

**Discussion:**

Definitive data are lacking to guide the clinical use of post-ED visit multidose ondansetron in children with acute gastroenteritis. Usage is increasing, despite the absence of supportive evidence. The incumbent additional costs associated with use, and potential side effects such as diarrhea and repeat visits, create an urgent need to evaluate the effect and safety of multiple doses of ondansetron in children focusing on post-emergency department visit and patient-centered outcomes.

**Trial Registration:**

ClinicalTrials.gov: NCT03851835. Registered on 22 February 2019.

## Background

The annual burden of acute gastroenteritis (AGE) in the USA includes 17 million related episodes and 473,832 hospitalizations [1]. Although oral rehydration therapy is recommended for children with mild-to-moderate dehydration, it has historically been underused by emergency department (ED) clinicians who are more likely to choose intravenous over oral rehydration, especially when vomiting is a major symptom [2]. Based on evidence of efficacy, the antiemetic agent ondansetron is frequently used to promote the success of oral rehydration therapy in children with vomiting secondary to AGE [3, 4]. At present, more than 80% of physicians routinely administer antiemetics to children who fail an oral fluid challenge, regardless of specialty training and practice location (i.e., community versus academic) [4]. Their use appears to be maximal (87%) among physicians working in pediatric EDs [4].

A 2013 overview [5] identified four studies that evaluated the efficacy of single-dose oral ondansetron compared to placebo in children with gastroenteritis presenting to an ED for medical care in high-income countries. Ondansetron administration reduces hospital admission and intravenous rehydration rates but has no impact on ED revisits. However, among the subgroup of children who did revisit an ED, those administered ondansetron at the index visit were less likely to receive intravenous rehydration at the revisit [5].

Although many studies have evaluated the use of single-dose oral ondansetron, very few have focused on the use of multiple doses. In 2002, Ramsook et al. [6] conducted a single-center, double-blind, placebo-controlled trial involving 145 children. They allocated children to receive six doses of ondansetron versus placebo administered every 8 h for 2 days. In keeping with other single-dose studies, vomiting was reduced after the first dose; however, no benefits occurred following discharge. At the 24- and 48-h follow-up time points, the median number of vomiting episodes remained zero, with no statistically significant difference between groups. However, during the 48 h following ED discharge, children administered ondansetron had threefold more diarrheal episodes than those in the placebo group (mean 7.7 versus 2.3) and a higher ED revisit rate (5% versus 0%; *P* = 0.05).

In 2010, in the only other multidose study to date, Yilmaz et al. conducted a randomized trial in Turkey [7]. They provided 109 participants with ondansetron or placebo every 8 h for 24 h. Although ondansetron administration reduced the frequency of vomiting following discharge (mean of 1.7 versus 0.2 episodes over 24 h; *P* < 0.001), its administration was associated with a small increase in the number of diarrheal episodes (5.0 versus 4.3 episodes; *P* = 0.04). There was no between-group difference with regards to ED return visits (ondansetron, 13%; placebo, 14%; *P* = 0.85).

The results of these clinical trials are conflicting and limited. They focus on individual symptoms and outcomes without enabling a proper assessment of overall wellbeing. Nonetheless, multiple-dose ondansetron use following ED discharge is on the rise. Between 2010 and 2015, 36% of nearly 12,000 children discharged from a New York City pediatric ED with AGE were provided with a prescription for home ondansetron use [8]. However, children prescribed ondansetron were equally likely to revisit the ED (adjusted odds ratio 1.12, 95% confidence interval 0.92–1.33) and be admitted at a revisit (adjusted odds ratio 0.81, 95% confidence interval 0.51–1.27). Similarly, in a study that included 996 children with AGE treated in a pediatric ED in Minneapolis between 2012 and 2014, where 71% of eligible children were discharged with prescriptions for ondansetron, there were no reductions in 3- (5% in both groups; *P* = 0.75) and 7-day (6% among those provided with a prescription versus 5% among those not provided a prescription; *P* = 0.66) ED revisits [9].

Given the limited literature regarding multiple-dose ondansetron use after ED discharge, the associated costs to the health care system and families, and the potential downsides of treatment, the proposed DOSE-AGE (multi-DOSE oral ondansetron for pediatric Acute GastroEnteritis) comparative effectiveness study will inform a key decision in the treatment of children with AGE.

## Methods/design

### Hypothesis

The provision of additional doses of ondansetron following the administration of an initial dose in the ED will be associated with improved outcomes compared with the administration of a single dose of ondansetron followed by a placebo in children aged 6 months to 18 years who present to a pediatric ED with ≥3 episodes of vomiting due to AGE in the preceding 24 h.

### Study questions

#### Clinical efficacy

Clinical efficacy will be determined by comparing the following outcomes in the two study groups:

1. Is there a difference in the proportion of children who develop moderate-to-severe disease (Modified Vesikari Scale (MVS) score ≥9)?
2. Is there a difference in the: i) time until the last vomiting episode after enrollment; ii) frequency of vomiting; or iii) proportion who experience vomiting?
3. Is there a difference in the proportion of children who require an unscheduled health care provider visit?
4. Is there a difference in the proportion of children who require intravenous rehydration?
5. Is there a difference in satisfaction with care, as reported by caregivers?

#### Safety

Do children with AGE-associated vomiting who are provided with additional doses of ondansetron for as-needed home use following an initial dose in the ED experience adverse effects (e.g., increased number of diarrheal episodes, cardiac symptoms) compared to those given a placebo?

### Study design

DOSE-AGE is a randomized, placebo-controlled, double-blinded, six-center, pragmatic clinical trial being conducted in six Canadian pediatric EDs. We will randomize 1030 children at a 1:1 ratio to receive either 48 h of ondansetron oral solution (0.15 mg/kg to a maximum single dose of 8 mg) [10] or a matching placebo to be administered by the child’s caregiver as needed.

### Ethics approval

Research Ethics Board (REB) approval has been granted by the board of record at each participating study site: Alberta Children’s Hospital/University of Calgary, Children’s Hospital of Eastern Ontario, Centre Hospitalier Universitaire Ste-Justine/Université de Montréal, London Health Sciences Centre/Western University, Stollery Children’s Hospital/University of Alberta, and The Children’s Hospital of Winnipeg/University of Manitoba. Following consultation with Health Canada, it was determined that a Clinical Trials Application with Health Canada’s Therapeutics Directorate was not required for this study.

### Study population

The study population will consist of children aged 6 months to 18 years diagnosed as having an acute intestinal infection by the ED treating physician and administered ondansetron during their ED visit. The diagnosis of an acute intestinal infection is at the discretion of the treating ED physician. A range of terminologies that may reflect an acute intestinal infectious process are acceptable provided they meet all other eligibility criteria (e.g., viral illness, diarrhea, vomiting, upper respiratory infection, postinfectious gastroenteritis, antibiotic-associated diarrhea, toddler’s diarrhea, viral infection, enteritis, viremia, fever and bronchiolitis) [11].

#### Inclusion criteria

All of the following criteria must be met for inclusion in the study:

1. Aged 6 months to <18 years
2. Presence of ≥3 episodes of vomiting in the 24 h preceding the ED visit
3. Duration of vomiting and diarrheal symptoms <72 h
4. Minimum of one episode of vomiting within 6 h of eligibility screening
5. Minimum of one dose of ondansetron (oral or intravenous) administered during the ED visit

#### Exclusion criteria

Individuals who meet any of the following criteria will be excluded from participation in the study:

1. Bilious or bloody vomitus
2. Allergy to ondansetron or any serotonin receptor antagonist
3. Known allergic reaction to components of ondansetron or the placebo medication
4. History or family history of long-QT syndrome [12]
5. Presence of complex congenital heart disease
6. History or family history of cardiac arrhythmia
7. History of glucose-6-phosphate dehydrogenase deficiency
8. Concomitant use of any of the following:
  - QTc [12] prolonging medications (e.g., methadone, antidepressants, antiarrhythmics)
  - Medications known to cause torsades de pointes (e.g., macrolide antibiotics, apo morphine)
  - Medications that cause electrolyte abnormalities (e.g., high-dose corticosteroids, diuretics)
  - Serotonergic or neuroleptic medications (e.g., triptans, selective serotonin reuptake inhibitors, serotonin norepinephrine reuptake inhibitors, fentanyl, monoamine oxidase inhibitors, lithium)
  - Any 5-hydroxytryptamine (5-HT)_3_ receptor antagonist excluding ondansetron
9. Inability to complete follow-up
10. Previously enrolled in study

### Intervention

**Fig. 1:**
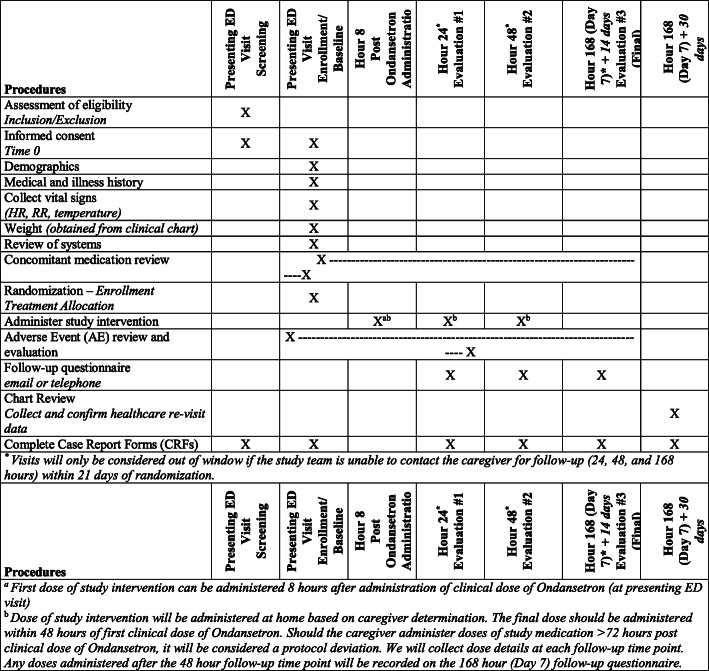
Schedule of activities. ED emergency department, HR heart rate, RR respiratory rate

The schedule of activities is shown in Fig. 1. Participants will be recruited from six Canadian EDs. Informed consent and assent, as per local guidelines, will be obtained from each participant and/or their legally authorized representative prior to study enrollment. The process includes approval for re-use of study data by approved researchers who have their new study approved by an ethics board and sign an agreement ensuring data confidentiality and the restriction of use to only the approved study.

Once consent has been obtained, the participant will be allocated, by a web-based randomization service (www.randomize.net) to receive either ondansetron oral solution or a matched placebo. The study medication dose will be 0.15 mg/kg, calculated using the participant’s weight as measured at the index ED visit. The maximal single dose that can be provided is 8 mg, which corresponds to 10 ml.

Caregivers will be instructed to administer a dose, based on need as assessed by the caregiver (i.e., for preverbal children) or expressed by the child (i.e., for verbal children), every 8 h to a maximum of three doses in a 24-h period. A dose should only be administered if it is felt the child is nauseated or experiencing ongoing vomiting and hence would be beneficial. Caregivers will be given a study medication diary to track the administration of study medication doses and a discharge instruction sheet explaining the primary benefits of medication use, how to contact the study team, study requirements, and health care provider follow-up criteria. If admitted to hospital, participants will continue on the study protocol. Enrolled children should not receive any nonstudy intervention doses of ondansetron during the 48-h period following randomization.

### Investigational agents

The active intervention administered is ondansetron 4 mg/5 ml oral solution (Zofran®; purchased from Sandoz Canada, Novartis Pharmaceuticals). Ondansetron hydrochloride is a selective serotonin receptor antagonist of the 5-HT_3_ subtype [13]. Its precise mode of action in the control of nausea and vomiting is unknown. A placebo solution, matched in taste, appearance, consistency and smell, was developed by the College of Pharmacy, University of Manitoba. The study intervention is dosed at 0.15 mg/kg (0.1875 ml/kg) to a maximum single dose of 8 mg [10]. Placebo stability testing was performed resulting in solution stability of 120 days from the compound date. Kits will be replaced prior to the expiry date and multiple batches will be employed at each study site.

### Randomization

To ensure allocation concealment, an independent, third-party web-based randomization service, www.randomize.net, was used to produce the study treatment allocation lists stratified by study site and weight (<20 kg versus ≥20 kg). The treatment allocation list was sent to the coordinating pharmacy at the Alberta Children’s Hospital. The research pharmacy team at the Alberta Children’s Hospital securely distributed the randomization list to each study site pharmacy where the study pharmacist prepared consecutively numbered kits according to the randomization schedule. The available kits are stored in site pharmacies or in the enrolling site’s ED. Kit inventory is temperature monitored and securely stored.

#### Allocation concealment

The web-based randomization system, www.randomize.net, utilizes industry-standard security to send data via the internet. The randomization has been designed to employ random blocks of 4 and 6 and to use a 1:1 allocation ratio. Stratification by site and weight and block randomization will ensure variables are comparably distributed across treatment arms. Randomize.net has the advantage of assigning the subject to a kit ID number during the actual randomization process (i.e., we will employ remote computer subject randomization, kit IDs are not sequentially assigned).

Unblinding should only occur when the treating physician and study investigator determine that knowledge of treatment allocation will alter the clinical care being provided. Approval for unblinding must be obtained from the principal investigator (SBF) or site investigator prior to unblinding treatment. If the principal investigator cannot be reached, in urgent situations unblinding can be performed by the site investigator and the principal investigator informed within 24 h via email or telephone call.

#### Implementation

A log of all screened patients will be maintained. If eligible, the details of the study will be discussed with the caregiver and patient. Once consent has been obtained, enrolled participants will be assigned a participant ID number by the clinical site via the study’s secure web-based software platform, REDCap (Research Electronic Data Capture) [14, 15]. REDCap provides: 1) an intuitive interface for validated data capture; 2) audit trails for tracking data manipulation and export procedures; 3) automated export procedures for seamless data downloads to common statistical packages; and 4) procedures for data integration and interoperability with external sources. Study staff will collect baseline demographic variables, clinical values, and current illness history data using direct data entry into REDCap via an electronic tablet. Subsequently, the participant will be randomized using www.randomize.net to obtain treatment allocation.

### Bias

The ondansetron and placebo oral solution are identical in appearance, consistency, taste, and smell. The solutions are packaged in identical, labelled, amber-colored glass bottles. Study staff, participants, caregivers, health care providers, data collectors and data analysts will remain blinded to treatment allocation until the end of the study. The trial was registered at www.clinicaltrials.gov prior to study initiation.

### Concomitant medications

Patients currently taking QTc-prolonging medications, medications known to cause torsades de pointes, medications that cause electrolyte abnormalities, serotonergic or neuroleptic medications, as well as any 5-HT_3_ receptor antagonists will be excluded from the study [13]. The use of concomitant medications is at the discretion of the child’s treating physician; however, the administration of ondansetron after randomization is not permitted. All concomitantly used medications will be recorded.

### Outcomes

#### Primary outcome

The primary outcome is the development of moderate-to-severe disease in the 7 days after the index ED visit as measured by the MVS score (Table 1) [16, 17]. Despite limited evidence describing the characteristics of the original 20-point Vesikari Score, it has been employed as a dichotomous variable in many clinical studies [19, 20]. The score correlates with other meaningful measures such as caregiver anxiety, helplessness, stress [21], parental worry, behavioral changes and impact on the parents’ daily activities and distress [22]. Challenges with the use of the original score in large outpatient trials, particularly with assessing percent dehydration—an element of the original score [11]—led to the development and validation of the MVS [16, 17]. The modified score includes an important and easy to obtain outcome that reflects global gastroenteritis disease severity: the need for unscheduled health care provider visits. This is supported by evidence that the utilization of professional medical care correlates with disease severity [21]. Recently, two large multicenter ED-based randomized controlled trials have employed the MVS as a primary outcome [23, 24].

**Table 1:** Modified Vesikari Scale score^a^

**Scale component**	**Score**			
	**0 Points**	**1 Point**	**2 Points**	**3 Points**
Diarrhea duration (h)	0	<96	96 to <120	≥120
Maximum number of watery stools/24-h period	0	1–3	4–5	≥6
Vomiting duration (h)	0	<24	24 to <48	≥48
Maximum number of vomiting episodes/24-h period	0	1	2–4	≥5
Maximum recorded rectal temperature (°C)^b^	<37.0	37.0 to <38.5	38.5 to <39.0	≥39.0
Unscheduled health care visit	None	NA	Primary care	Emergency department
Treatment	None	Rehydration with intravenous fluids	Hospitalization	NA

*NA* not applicable

^a^In the Modified Vesikari Scale score, one variable (percent dehydration) in the original score was replaced with the variable of unscheduled health care visits to better measure the effect of acute gastroenteritis in outpatients given that the ability to perform frequent in-person assessments in an outpatient cohort of children can be challenging; scores range from 0 to 20, with higher scores indicating more severe disease; children with a score of 9 or more were considered to have moderate-to-severe gastroenteritis [16, 17]

^b^Temperatures are adjusted for the location of measurement: 1.1°C was added to axillary temperatures and 0.6°C was added to oral temperatures [18]

Follow-up will occur, either by telephone or emailed survey (to be selected by the participant), at 24, 48 and 168 h (i.e., 7 days) after the index visit. After follow-up data have been collected, the MVS score will be calculated based on the information provided. Each variable in the MVS is assigned a score for the entire study period (time 0 to day 7), with each participant being assigned a single score for the study period. Participants are being asked to submit daily information (at 24 and 48 h) and then summative information (covering a 5-day period) based on our extensive experience that such an approach maximizes accuracy while minimizing burden and conflicting data [23, 25, 26]. Variables are scored based on the worst 24-h period (maximal frequency components), on the total duration of symptoms (duration components, in hours), or on the occurrence of an outcome (event components). While the data collected at baseline will allow us to calculate an index visit (i.e., pre-enrollment) MVS score, all participant scores will revert to 0 (i.e., no symptoms) following enrollment. The pre-enrollment score will serve as a covariate in a regression analyses and will be employed for subgroup analysis purposes. Only outcomes and symptoms that occur after study enrollment will contribute to the calculation of the primary outcome.

In the MVS derivation study [17], construct validity was proven by using scores of ≥9 to define moderate and ≥11 to define severe disease. These cut-off points were associated with significant increases in other measures of disease severity (e.g., daycare absenteeism (*P* = 0.01) and work absenteeism (*P* = 0.002)) [17].

#### Secondary outcomes

1. Frequency of vomiting from study enrollment to day 7. The total number of episodes of vomiting and diarrhea related to the presenting illness will be compared between groups. These data will be obtained from the study questionnaires performed at 24, 48 and 168 h after enrollment. Questionnaires will contain questions regarding vomiting presence, number of episodes and vomiting cessation date and time. Frequency will be defined as the total number of episodes across the study duration.
2. Duration of vomiting, defined as the total number of days the participant experiences vomiting from day 0 to day 7. These data will be obtained from the study questionnaires performed at 24, 48 and 168 h after enrollment. A participant with a 24-h break in symptoms will be assumed to have ceased vomiting.
3. Any vomiting within 7 days of enrollment. The proportion of participants will be determined using data from the participant questionnaires performed at 24, 48 and 168 h after enrollment.
4. Proportion of participants who have an unscheduled health care provider visit within 7 days of enrollment. Return visits for unscheduled care to a health care provider related to vomiting, diarrhea, dehydration, fever, abdominal pain or fluid refusal within 7 days, not including scheduled visits (e.g., reassessments, vaccinations, and so forth) will be assessed using follow-up questionnaires at 24, 48 and 168 h after enrollment. Return-to-care data will be confirmed by reviewing the participant’s hospital chart. Not all data within a patient’s medical record will be available to the research team; therefore, the questionnaires will be the primary source of these data.
5. Proportion of participants who require intravenous rehydration. If the participant required an unscheduled health care visit (see point 4 above), the participant will be asked additional questions related to intravenous insertion, treatment course and disposition. Intravenous fluids administered at the index visit will not be included as part of this outcome.
6. Caregiver satisfaction as measured by a five-point Likert scale in the 48 h following ED disposition. Caregiver satisfaction with the therapy provided (oral ondansetron or placebo) in the 48 h following ED disposition will be measured using a five-point Likert scale.

#### Safety outcomes

To determine if discharged children, with vomiting secondary to AGE who are administered ondansetron in the ED with additional doses taken at home as needed, are at increased risk of adverse events (AEs) compared with those receiving placebo, groups will be compared regarding the development of any side effects. We will also specifically report the frequency of diarrheal episodes during the 48 h following ED disposition and the maximal number of diarrheal episodes in a 24-h period [6, 25].

#### Data retrieval

Study data will be collected at the following time points using email or telephone communication:

1. Telephone and electronic survey communication 24, 48 and 168-h after the index visit. At the index visit, caregivers will select their preferred method of communication—email (electronic) or telephone. Caregivers will be contacted as scheduled by the identified method. Follow-up surveys will be performed using a standardized data collection form. Detailed questioning will follow positive responses, especially as it relates to symptoms of vomiting and diarrhea. Caregivers will report if, and how many, doses of the study medication were administered.
2. Medical chart review. Data regarding revisits, intravenous hydration and hospitalization will be verified by reviewing medical charts (electronic and/or paper) of enrolled participants using each study site’s medical record database.

#### Sample size estimates

The sample size estimate was based on the assessment of the between-group difference in proportions of children with a postrandomization score ≥9 on the MVS. The adoption of multidose, post-ED ondansetron use can be recommended if the proportion of the primary outcome is significantly lower among those who receive multidose ondansetron. Calculations were based on a two-sided α of 0.05 and power of 0.90. The null hypothesis is *H*_0_ : *P*_*C*_ − *P*_*I*_ = 0, where *P*_*I*_ and *P*_*C*_ are the outcome probabilities in the intervention and control groups, respectively. The alternative hypothesis is *H*_1_ : *P*_*C*_ − *P*_*I*_ ≠ 0.

Our estimated event rate in the control group emerges from the analysis of data from over 1000 children where ondansetron home use was rare. Participants were followed prospectively and approximately 30% of these children experienced moderate-to-severe disease following the index visit [27, 28]. Expert surveys have indicated that an absolute risk reduction of 10% in the occurrence of moderate-to-severe AGE following the index visit would constitute a minimal clinically important difference [23]. Therefore, our sample size calculation assumed a 30% event rate in the control group for which we desire to detect an absolute beneficial treatment effect of 10% with 90% power. Using a two-sided type I error of 0.05 and the hypothesized proportions yields a required total sample size of 784 patients [29]. Our expected power, should we find different event rates in our two groups, is displayed in Table 2.

**Table 2:** Power table based on 784 randomized children completing follow-up

**Outcome control**	**Outcome intervention**	**% Difference**	**Power**
0.25	0.15	0.10	0.94
0.25	0.20	0.05	0.39
0.25	0.25	0.00	NA
0.30	0.15	0.15	0.999
0.30	0.25	0.05	0.35
0.35	0.15	0.20	~1
0.35	0.20	0.15	0.997
0.35	0.25	0.10	0.86

*NA* not applicable

Based on previous work by our group [23, 30, 31], we assume a 10% loss to follow-up, 5% withdrawal, and 3% drop-in rate (i.e., provision of ondansetron outside of the study protocol). Thus, the final sample size required is 1030 participants.

### Study populations

#### Screening population

The screening population includes all patients who are screened for eligibility into the trial, regardless of randomization or treatment status. This population represents all patients who meet all inclusion criteria and who are screened in real time by study staff at the participating site. This population will be used for reporting study flow as per CONSORT guidelines.

#### Intention-to-treat population

The intention-to-treat (ITT) population includes all participants who are randomized into the trial regardless of adherence to the protocol, including, for example, participants who receive no study drug. The ITT population will be used for the primary efficacy analyses in the study and the main efficacy analyses of secondary outcomes. All analyses using the ITT population will be based on each participant’s assigned treatment arm, regardless of treatment actually received.

#### Safety analysis population

The safety analysis population will include a subset of participants who received any doses of the study medications. A subanalysis will be performed including only those who took three or more doses (the multidose population) to assess the impact of multiple doses of ondansetron on diarrhea and other safety outcomes. Reporting of results will be summarized according to treatment received. This population will be used for analysis of AEs to examine safety outcomes.

### Statistical analysis

All analyses will be completed using the ITT principle. AEs will use the ‘as-treated’ principle and a secondary safety analyses will employ the safety analysis population. All statistical tests will be two sided with significance declared at the 0.05 level. The family-wise error rate will be controlled for secondary outcomes using the Holm stepdown procedure [32]. All outcomes and baseline characteristics will be reported by study group, using frequency counts and percentages for discrete variables, and means, medians, standard deviations and interquartile ranges for continuous variables. A detailed statistical analysis plan is being concurrently published.

The primary analysis of the primary outcome will compare the proportion of children who develop moderate-to-severe disease utilizing a Mantel–Haenszel test, stratified by clinical center and weight category. For this analysis, we will report the absolute risk difference and the Mantel–Haenszel test statistic, alongside 95% confidence intervals. The secondary outcomes vomiting duration and frequency, proportion with vomiting, unscheduled health care provider visits, intravenous rehydration and caregiver satisfaction will be tested for between-group differences using appropriate tests. An additional exploratory analysis will assess the effects of treatment after adjusting for baseline covariates.

#### Safety

The proportion of children experiencing AEs, an aggregate outcome across all AEs, as reported by the caregivers, will be compared between groups using the Mantel–Haenszel test, stratified by site and weight strata. A secondary analysis will assess AEs in the multidose population. The frequency of diarrheal episodes during the 48 h following the index visit and the maximal number of diarrheal episodes in a 24-h period will be subject to statistical testing. No adjustment for multiplicity will be performed. Specific symptoms (e.g., palpitations, syncope) will be reported in frequencies by group and not subjected to hypothesis testing.

#### Planned subgroup analyses

The following prespecified subgroup analyses of the primary outcome will be performed:

1. Sex: male, female
2. Age: 6 months to <3.0 years, 3.0 to <6.0 years, 6.0 to <10.0 years, and ≥10.0 years
3. Vomiting frequency: ≤10 versus >10 episodes in the 24 h preceding study enrollment
4. Presence of diarrhea in 24 h preceding enrollment: yes, no

A subgroup effect will be declared significant if the interaction between treatment and the subgroup factor is significant in the appropriate statistical model for each interaction at a significance level of 0.05/4 = 0.0125.

### Data access

All deidentified individual participant data (including data dictionaries) will be made available immediately following publication with no end date to investigators whose proposed use of the data has been approved by an independent review committee identified for this purpose. Additional documents that will be available include the study protocol, statistical analysis plan and the informed consent forms. Proposals should be directed to stephen.freedman@ahs.ca. To gain access; data requestors will need to sign a data access agreement.

### Safety

Safety oversight will be under the direction of the Data and Safety Monitoring Board (DSMB), which is composed of individuals with expertise in pediatric emergency medicine, trial methodology and biostatistics. DSMB members include Drs Garth Meckler (Chair, Vancouver, Canada), Mark Roback (Minneapolis, USA), Anupam Kharbanda (Minneapolis, USA), Eyal Cohen (Toronto, Canada) and Lise Nigrovic (Boston, USA). Meetings will be held biannually and on an ad hoc basis.

The DSMB has had initial meetings and reviewed and approved the study protocol and monitoring plan; no interim analyses are currently requested by the DSMB. Should an interim analysis be required, it will be completed in accordance with the DSMB charter under the direction of the chair. Appropriate adjustments to the statistical analysis plan and significance level will be made if performed. Any analyses that are undertaken will be partially blinded with biostatisticians aware of the treatment arm but not the associated identifier (i.e., active or placebo). All by-treatment interim analyses will refer to arms as “A” and “B” throughout analysis and reporting to the study team and DSMB. The DSMB has the option to unblind treatment at any time should they deem it necessary. Early stopping for efficacy and futility will not be considered due to concerns for overestimating or underestimating treatment effects [33, 34].

Safety outcomes will be obtained via participant follow-up questionnaires performed 24, 48 and 168 h after enrollment. Summaries of incidence rates, intensity and relationship to study by system organ class and preferred term (MedDRA) [35] will be prepared and reported to the DSMB. All AEs beginning after randomization will be included.

An AE has been defined as any adverse occurrence in the health of a clinical trial participant which may or may not be caused by the administration of the study drug during the study period. Disease-related events are expected events considered to be part of the natural history of the underlying disease process of AGE and will not be reported as AEs. We have defined the following as disease-related events:

1. Hospitalization for intravenous rehydration or gastroenteritis-like symptoms
2. Future health care provider visits, including ED return visit for vomiting, diarrhea or dehydration
3. Intravenous rehydration
4. Abdominal pain, distention, bloating
5. Vomiting, diarrhea, fever, flatulence

Additionally, any symptom or sign that was present prior to randomization and that has not worsened since participation in the study began will not be reported as an AE.

Any serious adverse events (SAEs) that occur after randomization will be reported to the REB as appropriate and the study participant will be followed until satisfactory resolution or until the site investigator deems the event to be chronic or the participant is stable. An SAE has been defined as any of the following:

1. Medically significant; based upon appropriate medical judgment, the event may jeopardize the patient and may require medical or surgical intervention to prevent one of the other SAE-defined outcomes.
2. Congenital malformation or birth defect
3. Persistent or significant disability or incapacity
4. Life threatening; an event in which the patient was at immediate risk of death
5. Death

Participants may be withdrawn from the study by the site or principal investigator for the following reasons:

1. If any clinical AE, laboratory abnormality or other medical condition or situation occurs such that continued participation in the study would not be in the best interests of the participant
2. If the participant meets an exclusion criterion that precludes further study participation (e.g., safety concerns).
3. The participant’s caregiver wishes to withdraw their child for whatever reason.

Discontinuation from the study intervention does not indicate discontinuation from the study. Because caregivers will administer the study intervention on an as-needed basis, the study intervention may be discontinued at any time by the caregiver. It is not mandatory to administer all doses provided; therefore, the study protocol will not change unless discontinuation is due to an AE.

### Ancillary and post-trial care

No provisions have been made to provide ancillary, post-trial care or compensation to those who suffer harm from trial participation.

### Health economic analysis

An analysis of the cost effectiveness of multidose oral ondansetron will be undertaken to assess whether the intervention presents value for money as well as clinical effectiveness. A trial-based economic evaluation will be conducted and will conform to guidelines for economic evaluation from the Canadian Agency for Drugs and Technologies in Health [36]. The reference case will use a publicly funded health care payer perspective, with patient and societal costs considered in secondary analyses. The time horizon will be the first 7 days following discharge from the ED, as differences are only expected over this short time period.

Cost components in the reference case analysis will consist of the number of doses of ondansetron provided during presentation at the ED, admission to hospital or use of intravenous hydration after presentation at the ED, and use of other health services (e.g., family doctor, revisit to ED, hospital admission) within 7 days of enrollment. Additional cost components that will inform the patient and societal perspective analyses will include the number of doses of ondansetron provided under prescription, drug insurance coverage, transport and child care associated with health care use, and caregiver productivity losses. Health utilities will not be collected as many participants will be under 24 months of age and no reliable instruments are available for populations of this age. Due to this, the primary outcome of the trial, the MVS score, will be used as the primary outcome in the economic analyses.

Cost and outcome data will be combined to calculate an incremental cost-effectiveness ratio and a net monetary benefit statistic for the reference case and other analyses. A nonparametric bootstrapping approach will then be used to determine uncertainty surrounding point estimates and cost-effectiveness acceptability curves will be generated to communicate the likelihood that interventions are cost effective at a range of thresholds. If sufficient data are available, subgroup analysis will examine how cost effectiveness varies based on sex, age, and vomiting and diarrhea frequency in the 24 h before ED presentation.

### Discrete choice experiment and caregiver preferences for treatment outcomes

In conjunction with the pragmatic randomized controlled trial, a discrete choice experiment (DCE) will be conducted to examine caregiver preferences for different outcomes during treatment of AGE and the impact of this on the trial findings. The DCE approach asks participants to choose or trade-off between different scenarios with varying characteristics and, in doing so, allows estimation of the relative importance of different characteristics and, through that, their preferences to achieve differing treatment outcomes [37]. The DCE in this project will focus on the MVS score [16, 17] as the primary outcome measure in the trial, and will ask participants to make a series of choices between scenarios based on possible outcomes from the MVS score. This will allow an assessment of caregivers’ preferences for differing outcomes and whether the current scoring approach for the measure reflects these preferences for the disparate components of the measure. This can inform discussions about whether a different weighting system should be considered and can examine whether the trial would return different conclusions if this novel scoring approach was used.

To do this, the components included in the MVS will be translated into attributes within the DCE (e.g., vomiting duration, maximum number of vomiting episodes per 24-h period) and the possible scores within each component will be translated into levels within the DCE (e.g., 1 to 24 h; 2 to 4 episodes). An experimental design using these attributes and levels will then be generated according to principles of statistical efficiency, while balancing this with the need to censor illogical combinations and provide computational efficiency for participants. The DCE will be embedded in a wider questionnaire including sociodemographic information, access to health care, and previous experiences with AGE and, after piloting, will be distributed to an existing cohort of caregivers of children with AGE who provided permission to be contacted to participate in additional research studies [38]. In the questionnaire, respondents will choose between a series of choice sets which present possible treatment outcomes with differing characteristics across the included attributes and levels.

A multinomial logit model will be used to estimate average preferences across the sample as a whole. If a sufficient sample size is available, mixed multinomial logit models will be used to assess heterogeneity of preferences, which may highlight differences between subgroups within the sample. Descriptive characteristics of the sample will be reported and the results of the DCE analysis will be expressed as utility coefficients, and these coefficients will be used to calculate a DCE-derived weighting system for the components contained within the MVS. This novel DCE-derived weighting system will then be used to conduct exploratory analyses, and the primary analyses of the DOSE-AGE trial will be replicated with this scoring system. The results of these exploratory analyses will be compared with the primary analysis described above to examine whether consideration of caregiver preferences has an impact on the findings of the trial. It is important, however, to be clear that a novel weighting system would need consultation with clinicians and further validation, and the exploratory analyses will not supersede the main analyses from the trial.

### Trial management

Data collection and entry is the responsibility of the clinical trials staff at the study site. Site investigators are responsible for ensuring accuracy and completeness of reporting. The Women and Children’s Health Research Institute (University of Alberta, Edmonton, Canada) will act as the data coordinating center (DCC) housing all study data and will be responsible for the provision of data collection tools and clinical data management services. The methodology core (University of Toronto, Toronto, Canada), led by AH and PP, will be responsible for data analyses as outlined in the published study statistical analysis plan. SBF takes overall responsibility for the DOSE-AGE study. Study data monitoring will be performed by the University of Alberta Quality Management in Clinical Research group after the first patient has been enrolled at each site and every 6 months thereafter. The DCC and Quality Management in Clinical Research group will actively monitor study data remotely.

This study is part of a collaborative approach to enable the conduct of pediatric clinical research in Canada. Under the KidsCAN-PERC Innovative Pediatrics Clinical Trials initiative [39], four separate clinical trials will be implemented sharing common resources and infrastructure. The DOSE-AGE steering committee is chaired by TK and includes other members of the KidsCAN-PERC Innovative Pediatrics Clinical Trials initiative.

### Dissemination policy

All results from the study, be they positive, negative or inconclusive, will be published in international scientific journals. The project leader will enforce publication. Furthermore, the results will be presented at national and international conferences. Additionally, the results of this study will be communicated directly to the participants and to the public in general through the daily press and social media.

### Protocol amendments

Any modifications to the protocol that may impact on the conduct of the study, potential benefit of the patient or may affect patient safety, including changes of study objectives, study design, patient population, sample sizes, study procedures or significant administrative aspects, will require a formal amendment to the protocol. Such an amendment will be approved by the relevant REBs prior to implementation. All participating sites will be notified of all amendments via email and during monthly team meetings. The principal investigator and/or his delegates assume responsibility for updating all relevant stakeholders.

### Confidentiality

Participant confidentiality and privacy are strictly held in trust by the participating investigators, their staff and the sponsor. This confidentiality is extended to cover the clinical information relating to participants. Therefore, the study protocol, documentation, data and all other information generated will be held in strict confidence. No information concerning the study or the data will be released to any unauthorized third party without the prior written approval of the sponsor.

All research activities will be conducted in as private a setting as possible. The study monitor, other authorized representatives of the sponsor, representatives of the REB or regulatory agencies may inspect all documents and records required to be maintained by the investigator, including, but not limited to, medical records and pharmacy records for the participants in this study. The clinical study site will permit access to such records.

The study participant’s contact information will be securely stored at each clinical site for internal use during the study. At the end of the study, all records will continue to be kept in a secure location for as long a period as dictated by the reviewing REB, institutional policies or sponsor requirements.

Study participant research data, which are for purposes of statistical analysis and scientific reporting, will be stored in the REDCap electronic data capture system at the Women and Children’s Health Research Institute (WCHRI) DCC at the University of Alberta. These data will not generally include the participant’s contact or identifying information. Rather, individual participants and their research data will be identified by a unique study identification number. Permission to store data at the WCHRI DCC is included in the informed consent.

WCHRI’s REDCap installation is housed in a secure data center at the University of Alberta Hospital and is behind the Faculty of Medicine & Dentistry’s firewall. Data is entered through a web-based interface using 128-bit SSL encryption. Login is via a username/password pair with additional two-factor authentication. Additional information is available in WCHRI’s privacy document (https://redcap.ualberta.ca/privacy.pdf).

Coordinators and investigators at each study site only have access to data relating to their own study participants. Study management at the lead site and WCHRI DCC staff have access to data for all study participants. This access is required to perform system management functions, data cleaning and analysis. Once the study database is locked and data has been extracted for analysis, read-only access to the study database will be granted to the principal investigator and/or their designate if requested.

Participant contact information is stored in the REDCap data capture system for the purposes of follow-up contact via email. The participant’s sex, date of birth, weight and date of ED visit are also stored in the REDCap database for the purposes of return visit validation, chart reviews, accurate age and study medication dose calculations. Participants/caregivers are informed of data collection and storage policies during the informed consent process.

## Discussion

All investigators involved in the study have acknowledged a position of equipoise in relation to the discharge antiemetic management of children with AGE-associated vomiting; they accept that there is currently insufficient robust clinical evidence defining the optimal use of ondansetron in this group of children.

This study aims to evaluate the provision, at discharge, of additional doses of ondansetron to children who have already received an initial dose of ondansetron in the ED as part of their standard care for AGE-associated vomiting. It is recognized that there is heterogeneity within the pediatric emergency medicine community regarding the use of post-ED ondansetron. This study will contribute to the understanding of the optimal treatment approach to children with AGE-associated vomiting who seek ED care.

The trial-based economic evaluation will allow an examination of the cost effectiveness of the use of post-ED ondansetron and will inform discussions on whether this adaptation to care provides value for money. Finally, the DCE conducted alongside the trial will allow an examination of caregivers’ preferences for differing treatment outcomes after presentation at the ED for AGE-associated vomiting. This will allow an assessment of whether the scoring approach used as the primary outcome measure of the trial reflects differing preference weights assigned to outcomes within the measure and will also provide a wider consideration of whether changes to management of AGE-associated vomiting are aligned with caregiver preferences and whether scoring a measure according to caregiver preferences alters the findings of the trial.

### Trial status

As of 29 February 2020, 132 children have been enrolled at the six study sites. The current protocol version is 3.0. Anticipated completion of recruitment is 31 May 2022.

## Data Availability

Not applicable as no datasets are included in this study protocol.

## Abbreviations

5-HT: 5-Hydroxytryptamine
AE: Adverse event
AGE: Acute gastroenteritis
DCC: Data coordinating center
DCE: Discrete choice experiment
DSMB: Data and Safety Monitoring Board
ED: Emergency department
ITT: Intention-to-treat
MVS: Modified Vesikari Scale
REB: Research Ethics Board
REDCap: Research Electronic Data Capture
SAE: Serious adverse event
WCHRI: Women and Children’s Health Research Institute
